# Predictors of True Early Recurrence in Patients Undergoing Radiofrequency Ablation for Hepatocellular Carcinoma

**DOI:** 10.1002/jgh3.70400

**Published:** 2026-04-03

**Authors:** Yi‐Hao Yen, Kwong‐Ming Kee, Chao‐Hung Hung, Chien‐Hung Chen, Tsung‐Hui Hu, Jing‐Houng Wang, Sheng‐Nan Lu

**Affiliations:** ^1^ Division of Hepatogastroenterology, Department of Internal Medicine Kaohsiung Chang Gung Memorial Hospital and Chang Gung University College of Medicine Kaohsiung Taiwan

**Keywords:** carcinoma, hepatocellular, radiofrequency ablation, recurrence

## Abstract

**Aims:**

To analyze risk factors of true early recurrence in patients undergoing radiofrequency ablation (RFA) for early‐stage hepatocellular carcinoma (HCC).

**Methods and Results:**

We enrolled 791 patients with newly diagnosed early‐stage HCC (i.e., within Milan criteria) and Child–Pugh class A liver disease undergoing percutaneous RFA. Survival analysis was performed using the Kaplan–Meier method with the log‐rank test. Cox proportional hazards analysis was used to identify prognostic factors associated with early recurrence (i.e., recurrence within 2 years after RFA). Early recurrence was identified in 270 (34.1%) patients. After excluding 33 patients with local tumor progression (LTP) within 2 years of initial RFA, 237 patients were classified as showing true early recurrence. Multivariate analyses showed that multiple tumors (hazard ratio [HR] = 1.526; 95% confidence interval [CI] = 1.143–2.039), alpha‐fetoprotein (AFP) level of ≥ 10 ng/mL (HR = 1.533; 95% CI = 1.163–2.021), tumor size per 10 mm increase (HR = 1.019; 95% CI = 1.002–1.036), model for end‐stage liver disease (MELD) score per one increase (HR = 1.024; 95% CI = 0.999–1.049), anti‐hepatitis C virus (anti‐HCV) positivity (HR = 1.635; 95% CI = 1.187–2.252), and being treated with antiviral therapy for HCV (HR = 0.614; 95% CI = 0.434–0.869) were associated with inferior 2‐year recurrence‐free survival (RFS). We constructed a predictive model with these variables. This model provided three risk strata for 2‐year RFS, low risk, medium risk, and high risk, with 2‐year RFS of 75%, 58%, and 35%, respectively (*p* < 0.001).

**Conclusions:**

We developed a risk prediction model to predict true early recurrence in patients undergoing RFA for early‐stage HCC.

## 1 Introduction

Radiofrequency ablation (RFA) is widely used to treat hepatocellular carcinoma (HCC) and is recommended as one of the curative treatments for early‐stage disease [1, 2]. However, the tumor recurrence rate of patients with HCC is high after RFA [3, 4, 5, 6].

HCC recurrence is generally classified into early or late using 2 years after resection or ablation as the cutoff time point based on the assumed mechanisms [7], i.e., early recurrence is assumed to result from pre‐existing intrahepatic metastasis, whereas late recurrence is regarded as de novo tumor growth due to underlying liver disease [8]. Therefore, the ideal candidates for adjuvant treatment would be those with a high risk of early recurrence.

Several modalities, including intra‐arterial therapies and systemic therapies such as tyrosine kinase inhibitors and immune checkpoint inhibitors, are tested in adjuvant therapies, but none of them offer a survival benefit. However, there are ongoing adjuvant therapy trials for patients with HCC undergoing RFA or hepatectomy [9].

Multiple studies have reported risk factors of early recurrence in patients undergoing RFA for early‐stage HCC [10, 11, 12, 13, 14, 15]. However, none of these studies excluded patients with local tumor progression (LTP) [10, 11, 12, 13, 14, 15], which represent residual tumors rather than true recurrence. Therefore, in this retrospective study, we aimed to exclude patients with LTP after initial RFA and analyze risk factors of true early recurrence. Our findings may guide future adjuvant therapy for HCC patients with high risk of early recurrence after being treated with RFA.

## 2 Patients and Methods

Data were extracted from the HCC registry database of our institution. The criterion for inclusion in this study was patients with newly diagnosed early‐stage HCC (i.e., within Milan criteria) and Child–Pugh class A liver disease undergoing percutaneous RFA. The diagnosis of HCC was according to guideline recommendations [1, 2]. The flowchart of patient enrollment is shown in Figure S1.

## 3 RFA Procedure

The procedure for RFA was described in detail in our previous study [16, 17]. All patients underwent ultrasound‐guided RFA. Contrast‐enhanced computed tomography (CT) or magnetic resonance imaging (MRI) scans were performed to assess treatment effects 1 month after RFA. Complete response (CR) was considered if no enhanced area was demonstrated at the site of the index tumor [18]. For patients without CR, repeat RFA or transarterial chemoembolisation (TACE) was performed until CR was achieved. After CR, all patients were followed up with serum alpha‐fetoprotein (AFP) testing plus ultrasound every 3 months. Contrast‐enhanced CT or MRI was performed when recurrence was suspected. Recurrence was classified into three categories: LTP, intrahepatic distant recurrence (IDR), and extrahepatic metastasis (EM). LTP was defined as the presence of enhancing tumor adjacent to the ablated area after achievement of CR. IDR was defined as the presence of tumor recurrence in locations not adjacent to the ablated area [19].

Overall survival (OS) was defined as the time elapsed between the date of treatment and the date of the last follow‐up or death. Patients who were lost to follow‐up were censored at their last medical visit. Recurrence‐free survival (RFS) was defined as the time elapsed between the date of treatment and the date of the last follow‐up or recurrence. Those who did not present with recurrence were censored on the last follow‐up. Recurrence was defined according to guideline recommendations [1, 2].

## 4 Antiviral Treatment History

Nucleos(t)ide analog therapy was indicated for chronic hepatitis B patients with hepatitis B surface antigen (HBsAg) positivity and serum HBV DNA > 2000 IU/mL with serum alanine aminotransferase (ALT) elevation, patients with cirrhosis, or patients undergoing curative treatments for HCC. Antiviral treatment was indicated for chronic hepatitis C patients with RNA‐detectable, anti‐HCV‐positive serum hepatitis C virus (HCV) infection. Pegylated interferon plus ribavirin was used before January 1, 2017. Oral direct‐acting antivirals (DAAs) were used after January 1, 2017.

## 5 Statistical Analyses

Data are presented as number (percentage) or median (interquartile range [IQR]). OS and RFS were compared between groups using the Kaplan–Meier estimator and log‐rank test. To obtain the optimal cutoff value for AFP to predict 2‐year RFS after RFA, the minimal *p‐*value approach was used [20]. All variables were included in multivariate analysis using a stepwise method. Patients with missing data were excluded from the multivariate analysis. A nomogram was constructed according to the multivariate analysis. The nomogram for predicting 2‐year RFS was based on proportionally converting each regression coefficient in the multivariate model to a point range of 0–100. Total points derived from all variables in the nomogram were converted to predicted probabilities [21]. In line with Royston et al., we categorized the model into four groups at the 16th, 50th and 84th centiles in our data [22]. The model's performance was evaluated using internal validation through the bootstrap method with 200 resamplings. Internal validation was chosen over splitting the sample to reduce the chance of generating models with suboptimal performance (i.e., models with unstable performance and the same performance as that obtained with half the sample size) [23]. The model was calibrated using calibration plots and by comparing predicted and observed survival curves. All *p*‐values were two‐tailed, and a *p* < 0.05 was considered statistically significant. All statistical analyses were performed using SPSS Statistics software (version 25; IBM Corp., Armonk, NY, USA).

## 6 Results

### 6.1 Characteristics of Patients With Early‐Stage HCC Undergoing RFA

In the cohort of patients in this study, 389 (49.2%) were aged > 65 years; 495 (62.6%) were male; 162 (20.5%) were with multiple tumors; 459 (58.0%) were with tumors > 20 mm in size; 289 (36.5%) were Barcelona Clinic Liver Cancer (BCLC) stage 0; 502 (63.5%) were BCLC stage A; 289 (36.5%) had an AFP level of ≥ 20 ng/mL; 330 (41.7%) were HBsAg positive; 376 (47.5%) were anti‐HCV positive; 378 (47.8%) were with HCCs diagnosed by pathology; 81 (10.2%) were with alcohol use disorder; 638 (80.5%) were cirrhotic; the median (IQR) body mass index was 25.2 (22.8–27.9); and the median (IQR) model for end‐stage liver disease (MELD) score was 8.2 (6.8–10.1). In this cohort, 261 patients were treated with antiviral therapy for HBV and 228 patients were treated with antiviral therapy for HCV.

### 6.2 5‐Year OS and RFS in This Cohort

**FIGURE 1:**
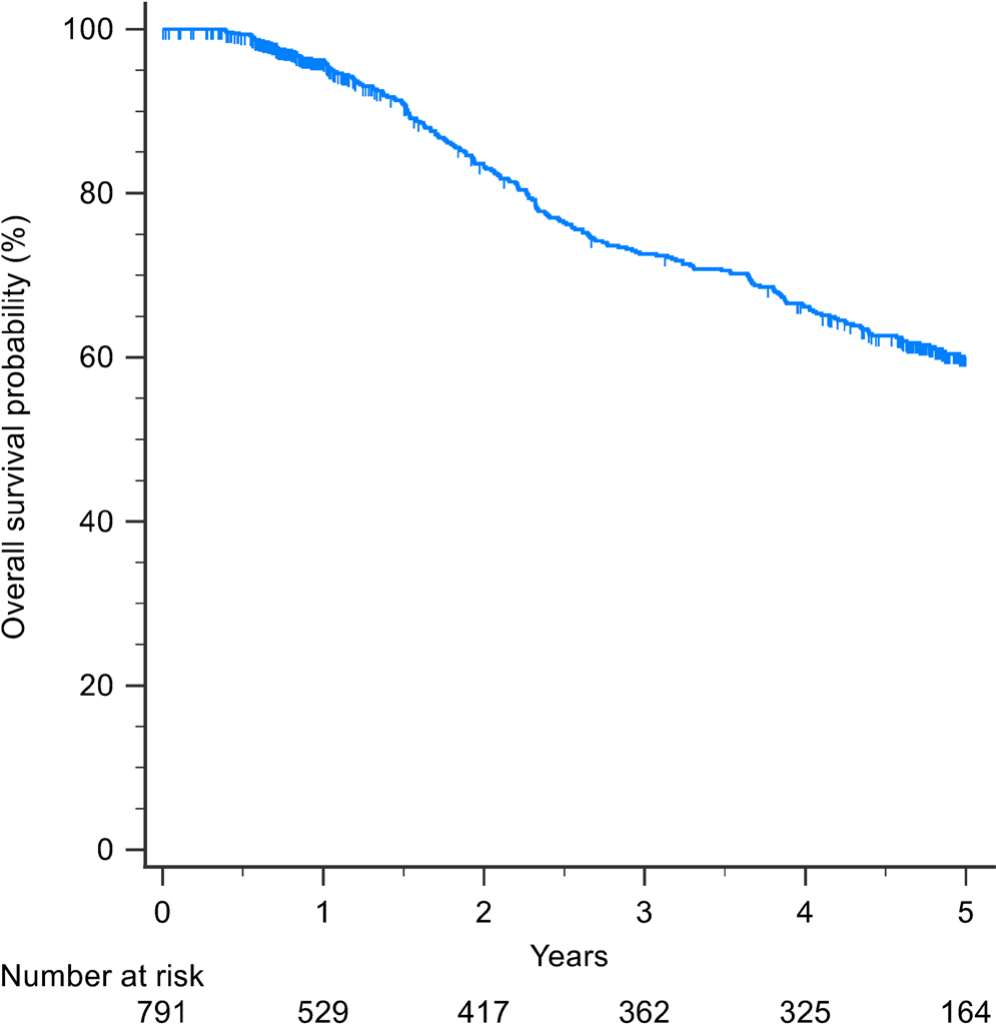
Kaplan–Meier curves for overall survival.

**FIGURE 2:**
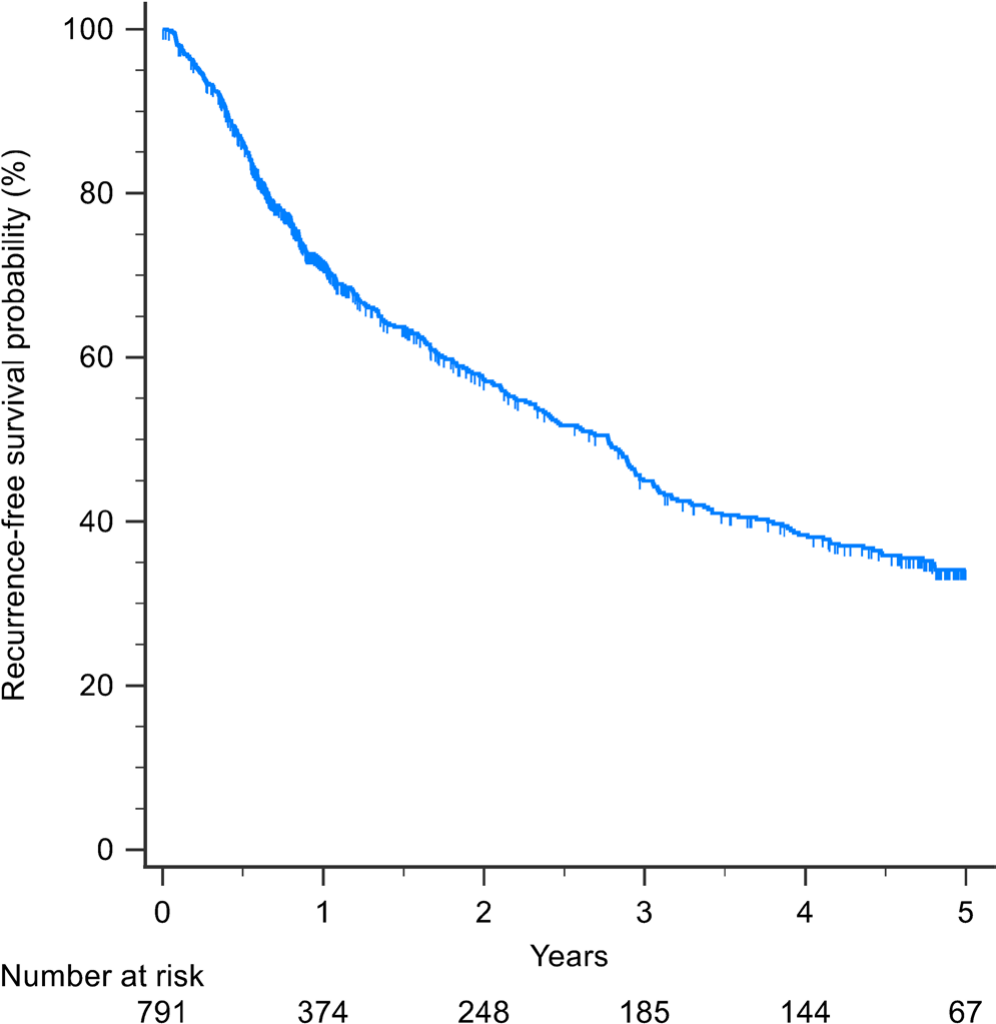
Kaplan–Meier curves for recurrence‐free survival.

The median (IQR) follow‐up period was 2.3 (0.9–4.9) years; 369 (46.6%) patients developed recurrence and 252 (31.9%) patients died. The 5‐year OS was 60% (Figure 1) and the 5‐year RFS was 34% (Figure 2).

### 6.3 Early Recurrence After RFA

Early recurrence (i.e., recurrence within 2 years of initial RFA) was identified in 270 (34.1%) patients. Of these 270 patients, 34 (12.6%) showed LTP, 228 (84.4%) showed IDR, and 8 (3.0%) showed EM. Of the 34 patients with LTP, 22 were recurrence‐free after repeat treatments for LTP, whereas the remaining 12 further developed IDR or EM after repeat treatments for LTP. Of the 12 patients showing IDR, recurrence occurred within 2 years of initial RFA only in one patient, while in the remaining 11 patients, recurrence occurred beyond 2 years from the initial RFA. In summary, of the 34 patients with LTP within 2 years of initial RFA, only one showed true early recurrence. Thus, 237 (30.0%) patients were classified as showing true early recurrence in the final analysis. The 2‐year RFS was 63% and 5‐year RFS was 37% in this cohort (Figure S2).

### 6.4 Cutoff Value of AFP to Predict 2‐Year RFS

We used the minimal *p*‐value approach to select the optimal cutoff value of AFP to predict 2‐year RFS. Commonly used cutoff values of AFP, including 10, 20, 100, 200, and 400 ng/mL, were selected to predict 2‐year RFS. The HR and *p*‐value of each cutoff are as follows: AFP ≥ 10 ng/mL, HR = 1.650 (95% CI = 1.269–2.145), *p* = 0.00019; AFP ≥ 20 ng/mL, HR = 1.572 (95% CI = 1.218–2.029), *p* = 0.00051; AFP ≥ 100 ng/mL, HR = 1.440 (95% CI = 1.052–1.972), *p* = 0.02294; AFP ≥ 200 ng/mL, HR = 1.466 (95% CI = 1.019–2.108), *p* = 0.03903; and AFP ≥ 400 ng/mL, HR = 1.103 (95% CI = 0.682–1.783), *p* = 0.69035. The 10 ng/mL cutoff for AFP was selected due to its *p*‐value being the lowest.

### 6.5 Univariate and Multivariate Cox Proportional Hazards Analyses of 2‐Year RFS

Univariate analyses showed that multiple tumors (HR = 1.579; 95% CI = 1.182–2.108; *p* = 0.002); AFP ≥ 10 ng/mL (HR = 1.666; 95% CI = 1.279–2.169; *p* < 0.001); MELD score per one increase (HR = 1.026; 95% CI = 1.002–1.050; *p* = 0.033); tumor size per 10 mm increase (HR = 1.022; 95% CI = 1.005–1.039; *p* = 0.011); HBsAg positivity (HR = 0.717; 95% CI = 0.548–0.938; *p* = 0.015); anti‐HCV positivity (HR = 1.370; 95% CI = 1.060–1.772; *p* = 0.016); and being treated with antiviral therapy for HBV (HR = 0.700; 95% CI = 0.523–0.937; *p* = 0.017) were associated with inferior 2‐year RFS (Table 1). After excluding 11 patients with missing data, the remaining 780 patients were included in multivariate analyses. The analyses showed that multiple tumors (HR = 1.526; 95% CI = 1.143–2.039; *p* = 0.004); AFP ≥ 10 ng/mL (HR = 1.533; 95% CI = 1.163–2.021; *p* = 0.002); tumor size per 10 mm increase (HR = 1.019; 95% CI = 1.002–1.036; *p* = 0.022); MELD score per one increase (HR = 1.024; 95% CI = 0.999–1.049; *p* = 0.059); anti‐HCV positivity (HR = 1.635; 95% CI = 1.187–2.252; *p* = 0.003); and being treated with antiviral therapy for HCV (HR = 0.614; 95% CI = 0.434–0.869; *p* = 0.006) were associated with inferior 2‐year RFS (Table 2).

**TABLE 1:** Univariate Cox proportional hazards analysis of 2‐year recurrence‐free survival.

	HR (95% CI)	*p*
Age > 65 years	0.972 (0.752–1.254)	0.825
Men	0.888 (0.684–1.152)	0.370
Multiple tumors	1.579 (1.182–2.108)	0.002
Tumor size per 10 mm increase	1.022 (1.005–1.039)	0.011
AFP ≥ 10 ng/mL	1.666 (1.279–2.169)	< 0.001
MELD score per one increase	1.026 (1.002–1.050)	0.033
HBsAg positivity	0.717 (0.548–0.938)	0.015
Anti‐HCV positivity	1.370 (1.060–1.772)	0.016
Being treated with anti‐viral therapy for HBV	0.700 (0.523–0.937)	0.017
Being treated with anti‐viral therapy for HCV	0.846 (0.636–1.126)	0.251

Abbreviations: AFP, alpha‐fetoprotein; CI, confidence interval; HBsAg, hepatitis B virus surface antigen; HBV, hepatitis B virus; HCV, hepatitis C virus; HR, hazard ratio; MELD, model for end‐stage liver disease.

**TABLE 2:** Multivariate Cox proportional hazards analysis of 2‐year recurrence‐free survival.

	*β* coefficient	HR (95% CI)	*p*
Multiple tumors	0.423	1.526 (1.143–2.039)	0.004
Tumor size per 10 mm increase	0.019	1.019 (1.002–1.036)	0.032
AFP ≥ 10 ng/mL	0.427	1.533 (1.163–2.021)	0.002
MELD score per one increase	0.024	1.024 (0.999–1.049)	0.059
Anti‐HCV positivity	0.491	1.635 (1.187–2.252)	0.003
Being treated with antiviral therapy for HCV	−0.488	0.614 (0.434–0.869)	0.006

Abbreviations: AFP, alpha‐fetoprotein; CI, confidence interval; HCV, hepatitis C virus; HR, hazard ratio; MELD, model for end‐stage liver disease.

### 6.6 Construction of a Nomogram to Predict Early Recurrence

**FIGURE 3:**
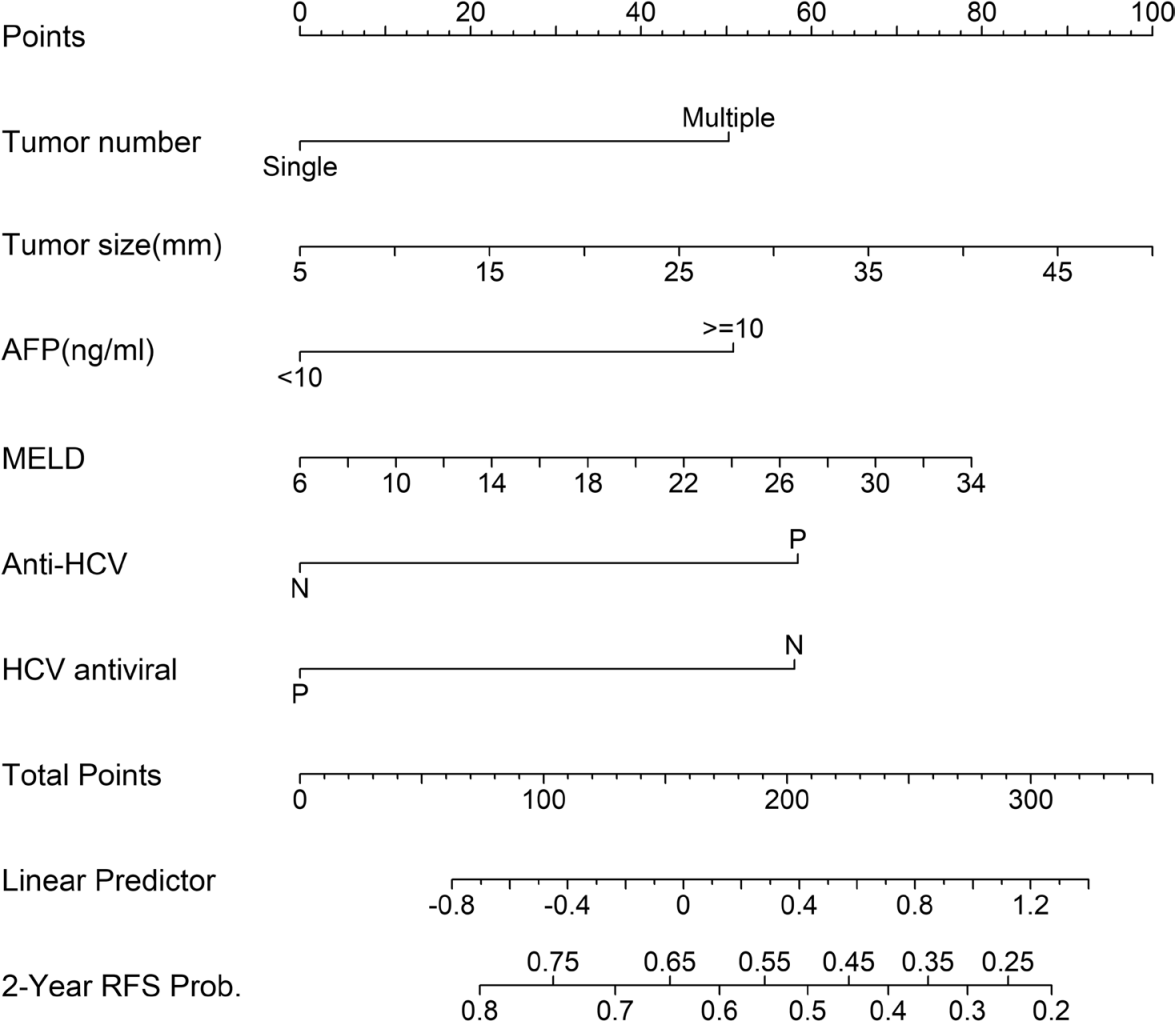
A nomogram for predicting early recurrence.

In this study, we identified six variables (AFP level, tumor number, tumor size, anti‐HCV positivity, being treated with antiviral therapy for HCV, and MELD score) for constructing a nomogram to predict early recurrence (Figure 3). An example is a patient with a single tumor (score = 0), tumor size of 25 mm (score = 45), AFP = 20 ng/mL (score = 50), MELD score = 10 (score = 10), and anti‐HCV positivity (score = 57.5) and in receipt of antiviral therapy for HCV positivity (score = 0). The total points for this patient were 162.5. We drew a vertical line from total points to 2‐year RFS probability. The 2‐year RFS of this patient was 0.62.

### 6.7 A Risk Stratification System for Predicting Early Recurrence

**FIGURE 4:**
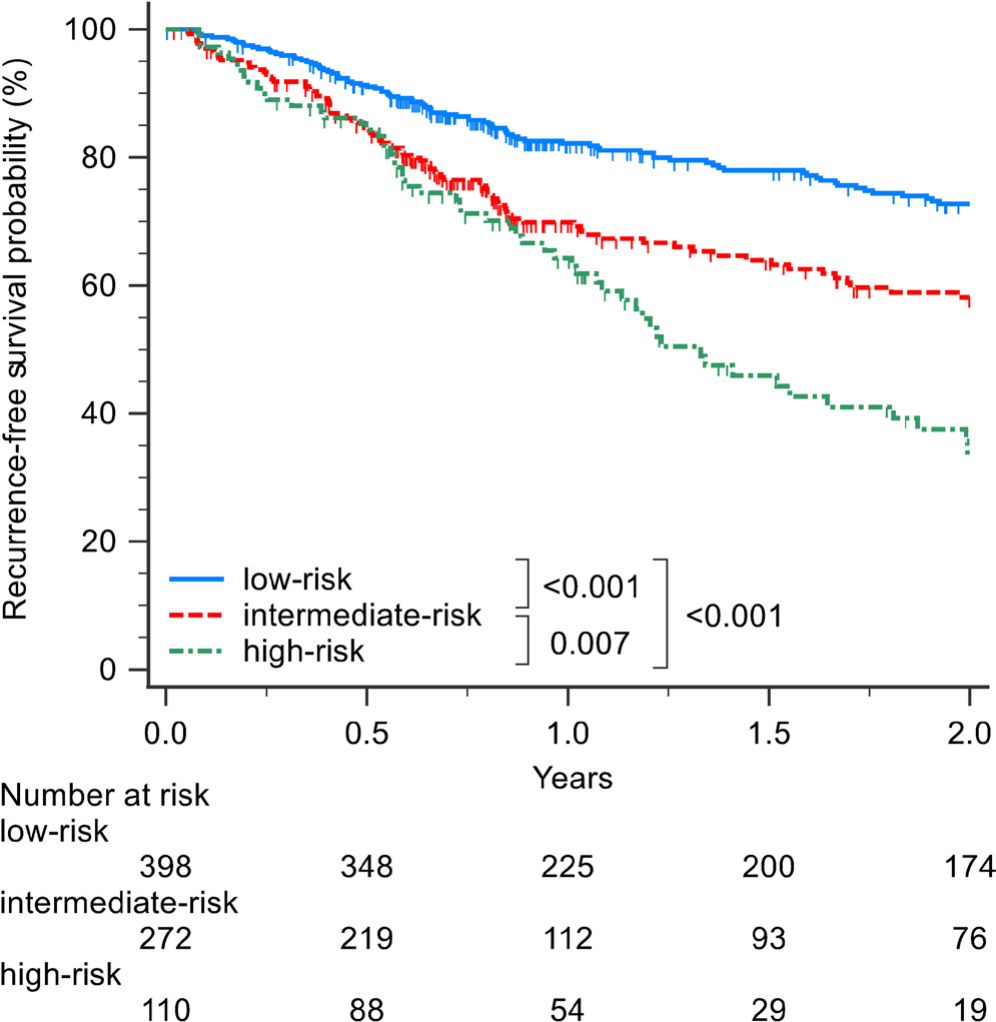
Kaplan–Meier curves for 2‐year recurrence‐free survival stratified by three risk groups.

The risk score was derived from the nomogram (Figure 3). To create prognostic groups, we categorized the model into four groups at the 16th, 50th, and 84th centiles of the estimated risk score based on early recurrence [22]: a very‐low risk group of patients with their risk scores below the 17th percentile; a low‐risk group of patients with their risk scores between the 17th and 49th percentile; a medium‐risk group with their risk scores between the 50th and 85th percentiles; and a high‐risk (maximum risk) group with their risk scores above the 85th percentile. The patient‐specific linear prediction was calculated and cutoff values were applied to classify patients into four prognostic groups: those with scores of 73.8–103.6 were classified as very low risk; those with scores of 103.7–152.6 as low risk; those with scores of 152.7–212.0 as medium risk; and those with scores of 212.3–322.4 as high risk. Their 2‐year RFS was 80%, 70%, 58%, and 36% (*p* < 0.001), respectively (Figure S3). 2‐Year RFS was significantly different between the very‐low‐risk and medium‐risk groups (*p* < 0.001), between the very‐low‐risk and high‐risk groups (*p* < 0.001), between the low‐risk and medium‐risk groups (*p* = 0.005), between the low‐risk and high‐risk groups (*p* < 0.001), and between the medium‐risk and high‐risk groups (*p* = 0.007). However, the very‐low‐risk and low‐risk groups did not differ significantly (*p* = 0.082; Figure S3). Consequently, we merged the very‐low‐risk and low‐risk groups into one group (designated as the low‐risk group). Patients with scores of 73.8–152.6 were classified as low risk (*n* = 398); those with scores of 152.7–212.0 as medium risk (*n* = 272); and those with scores of 212.3–322.4 as high risk (*n* = 110). During the follow‐up period, 89 (22.4%) patients showed early recurrence in the low‐risk group, 91 (33.5%) in the medium‐risk group, and 56 (50.9%) in the high‐risk group. Their 2‐year RFS was 75%, 58%, and 35% (*p* < 0.001), respectively (Figure 4). 2‐Year RFS was significantly different between the low‐risk and medium‐risk groups (*p* < 0.001), between the low‐risk and high‐risk groups (*p* < 0.001), and between the medium‐risk and high‐risk groups (*p* = 0.007). The calibration plots showed overall high agreement between the predictions made by the model and observed outcomes (Figure S4).

**FIGURE 5:**
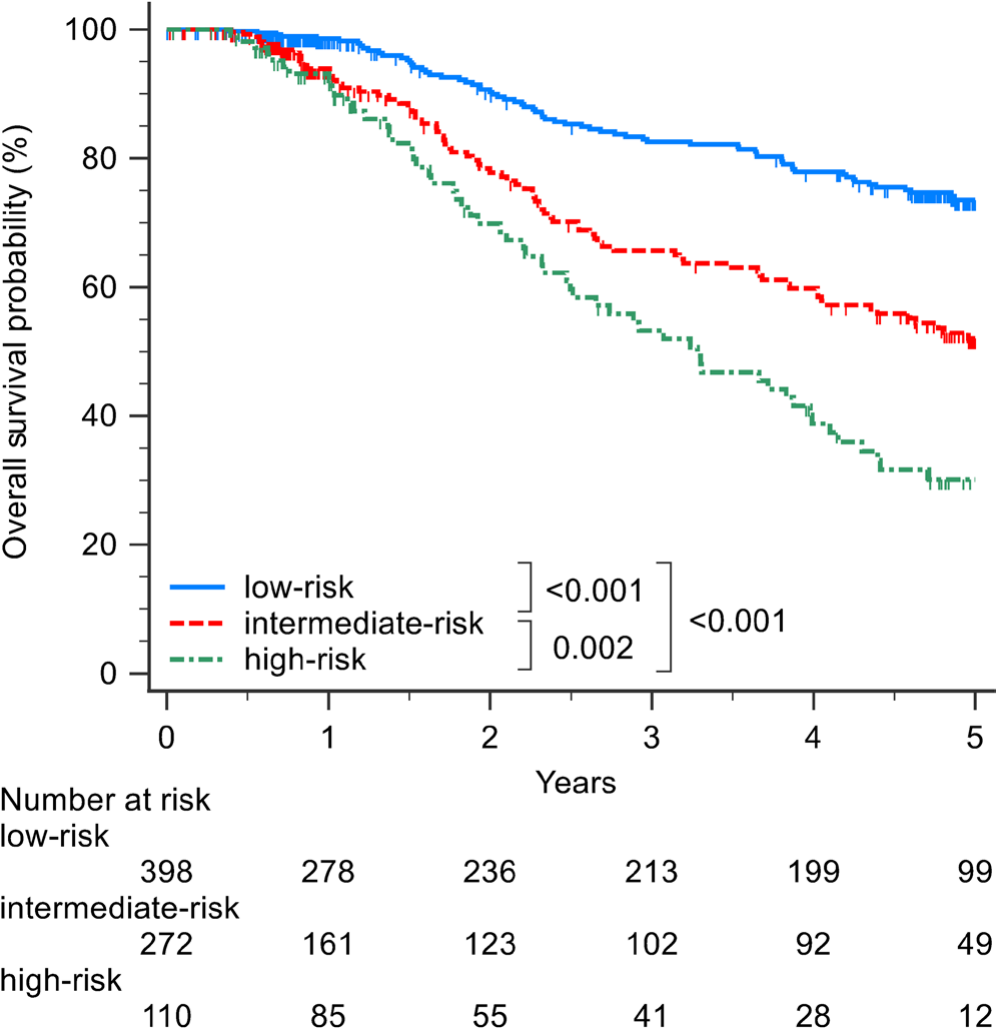
Kaplan–Meier curves for 5‐year overall survival stratified by risk groups.

During the follow‐up period, 86 (21.6%) patients died after RFA in the low‐risk group, 101 (37.1%) in the medium‐risk group, and 63 (57.3%) in the high‐risk group. This model also provided three risk strata for 5‐year OS: low risk, with 5‐year OS of 75%; medium risk, with 5‐year OS of 54%; and high risk, with 5‐year OS of 30% (*p* < 0.001). 5‐Year OS was significantly different between the low‐risk and medium‐risk groups (*p* < 0.001), between the low‐risk and high‐risk groups (*p* < 0.001), and between the medium‐risk and high‐risk groups (*p* = 0.002; Figure 5).

## 7 Discussion

The novelty of the present study is that we excluded patients with LTP within 2 years of initial RFA. Thus, we could analyze risk factors of true early recurrence in patients with HCC who underwent RFA. LTP is regarded as incomplete ablation rather than true recurrence. In a previous study of 103 patients with HCC treated with RFA, postrecurrence survival of those with LTP was the highest compared to the survival of those with IDR or recurrence with BCLC stage C (i.e., EM or portal vein tumor thrombosis) [24]. Wu et al. studied 274 patients receiving RFA for HCV‐related HCC. They found that HCV eradication was not associated with LTP but was significantly correlated with reduced risk of distant recurrence (HR = 0.449, *p* = 0.006) [25]. These studies support the concept that LTP is incomplete ablation rather than true recurrence [24, 25].

Tsuji et al. studied 112 patients with early‐stage HCC who underwent RFA. They excluded patients with LTP from their analyses. The 112 patients were divided into the IDR group (*n* = 51) and the no recurrence group (*n* = 61). Multivariate analyses showed that the neutrophil‐to‐lymphocyte ratio (NLR) (HR = 2.40; 95% CI = 1.44–3.99) and lens culinaris agglutinin a‐reactive fraction of AFP (HR = 1.02; 95% CI = 1.01–1.04) were independently associated with post‐RFA IDR. However, Tsuji et al. did not mention whether they included patients with EM after RFA in their analysis. In addition, their study included only a limited number of cases [26].

We developed a nomogram with six variables—AFP level, tumor number, tumor size, anti‐HCV positivity, being treated with antiviral therapy for HCV, and MELD score—to predict early recurrence. Internal validation with bootstrapping showed overall high agreement between the predictions made by the model and observed outcomes. Finally, this model could stratify patients into three groups based on true early recurrence. In addition, it could also stratify patients into three groups based on 5‐year OS.

Elevated AFP levels, increased tumor size, and multiple tumors are well‐known prognostic factors of patients with HCC [1, 2]. Late recurrence is regarded as de novo tumor growth due to underlying liver disease [8]. Therefore, poor liver function reserve is assumed to be associated with late recurrence. However, a higher MELD score was associated with early recurrence in the present study. A similar finding was noted in a previous study, which also showed that poor liver function reserve (i.e., higher albumin–bilirubin grade) was associated with early recurrence [12]. Multivariate analyses showed that anti‐HCV positivity and being treated with antiviral therapy for HCV were associated with 2‐year RFS. The possible explanations are as follows. Around 99% of patients could achieve a sustained virological response after DAA treatment [27]. However, DAAs have been reimbursed by the government only since January 1, 2017, and interferon‐based therapy was used before January 1, 2017. The poor tolerance and low sustained virological response rate of interferon‐based therapy [28] may explain anti‐HCV positivity being an independent risk factor of inferior 2‐year RFS. In contrast, nucleos(t)ide analog therapy is well tolerated [29], which could explain HBsAg positivity not being an independent risk factor of inferior 2‐year RFS. A previous study reported that DAAs improve recurrence rates after RFA or hepatectomy for early‐stage HCC [30], which may be the reason that being treated with antiviral therapy for HCV had a positive effect on 2‐year RFS.

The model in this study also provided three risk strata for OS—low risk, with 5‐year OS of 75%; medium risk, with 5‐year OS of 54%; and high risk, with 5‐year OS of 30% (*p* < 0.001). We believe that high‐risk patients may be ideal candidates for adjuvant treatment due to poor 5‐year OS; low‐risk patients may not benefit from adjuvant treatment due to satisfactory 5‐year OS, whereas the benefit for the medium‐risk group is debatable. Therefore, our model could be applied to clinical practice.

Although previous studies reported predictive models for early recurrence after RFA for HCC [10, 11, 12, 13, 14, 15], some of them had limited case numbers: *n* = 152 in Cha et al.'s study [12]; *n* = 108 in Ni et al. [13]; *n* = 543 in Xin et al. [11]; *n* = 279 in Yang et al. [10]; *n* = 90 in Zhang et al. [14]; and *n* = 186 in Liu et al. [15] Predictors of early recurrence in these studies included tumor‐related factors, i.e., tumor size [11], tumor number [10, 11, 13, 14], tumor markers [10, 11, 12, 13], and the presence of microvascular invasion (MVI) by radiomics [12, 13, 14, 15]; liver‐related factors such as albumin–bilirubin grade [12] and albumin [10, 14]; and inflammation‐based scores, such as NLR [13] and systemic inflammation response index (defined as neutrophil count × monocyte count/lymphocyte count) [11]. However, compared to these previous studies [10, 11, 12, 13, 14, 15], our study had the largest case number (*N* = 791).

This study has several strengths. First, its sample size is large. Second, it identified and excluded from early recurrence analysis patients with LTP within 2 years after the initial RFA, whereas previous studies did not [10, 11, 12, 13, 14, 15]. Third, a substantial proportion of its cohort consisted of patients with viral hepatitis. This study evaluated antiviral treatment history and its association with recurrence, whereas previous studies did not [10, 11, 12, 13, 14, 15]; the recurrence rates of patients with HBV differ depending on the use of nucleos(t)ide analog therapy [31], whereas those of patients with HCV differ depending on whether DAA therapy was administered [30]. The limitations of the present study are as follows. First, being a retrospective single‐center study, its results may not be generalizable to other institutions. Taiwan is a hepatitis B virus (HBV)‐endemic country; therefore, whether the results of the present study could be applicable to Western countries where HCV and alcohol use disorder are the leading causes of HCC is unclear. Other studies predicting early recurrence after RFA for HCC are also from East Asia, where HBV is the leading etiology of HCC [10, 11, 12, 13, 14, 15]. Second, the model lacks external validation. Previous studies reported that including image features suggestive of MVI may increase predictive accuracy of early recurrence [12, 13, 14, 15]; however, its drawbacks (e.g., lack of standardization in image acquisition schemes, varied reproducibility in extracting quantitative imaging features from tumor regions by different methods, etc.) limit its clinical translation [32].

## 8 Conclusion

We have developed a risk prediction model to forecast true early recurrence in patients with early‐stage HCC undergoing RFA. This model can be applied to ascertain patients with HCC who might benefit from adjuvant treatment.

## Funding

This study was supported by the Chang Gung Memorial Hospital‐Kaohsiung Medical Center, Taiwan (CORPG8N0271).

## Ethics Statement

The Institutional Review Board of Chang Gung Memorial Hospital–Kaohsiung Branch approved this study (reference number: 202201189B0).

## Consent

Informed consent was waived by the Institutional Review Board of Chang Gung Memorial Hospital–Kaohsiung Branch due to the retrospective nature of the study.

## Conflicts of Interest

The authors declare no conflicts of interest.

## Supporting information


**Figure S1:** Flowchart of patient enrollment.

**Figure S2:** Kaplan–Meier curves for recurrence‐free survival after excluding 33 patients who showed local tumor progression within 2 years of initial radiofrequency ablation.

**Figure S3:** Kaplan–Meier curves for 2‐year recurrence‐free survival stratified by four risk groups.

**Figure S4:** Calibration curves of the nomogram for predicting early recurrence.

## Data Availability

Raw data for the cohort involved in this study is available via the following digital object identifier: https://www.dropbox.com/scl/fi/ejlbd7yj3zaq25q1tmof5/20250606‐N791‐raw‐data‐for‐submission.xlsx?rlkey=bganpas79l89sesrka01angoa&st=osg94fus&dl=0.
