# Supplementary material for: Predictors of True Early Recurrence in Patients Undergoing Radiofrequency Ablation for Hepatocellular Carcinoma
Source: JGH Open. 2026 Apr 3;10(4):e70400. doi: 10.1002/jgh3.70400 (PMC13051943; doi:10.1002/jgh3.70400)
Supplement: Supplementary file 1 — Figure S1: Flowchart of patient enrollment. [file JGH3-10-e70400-s004.pdf]

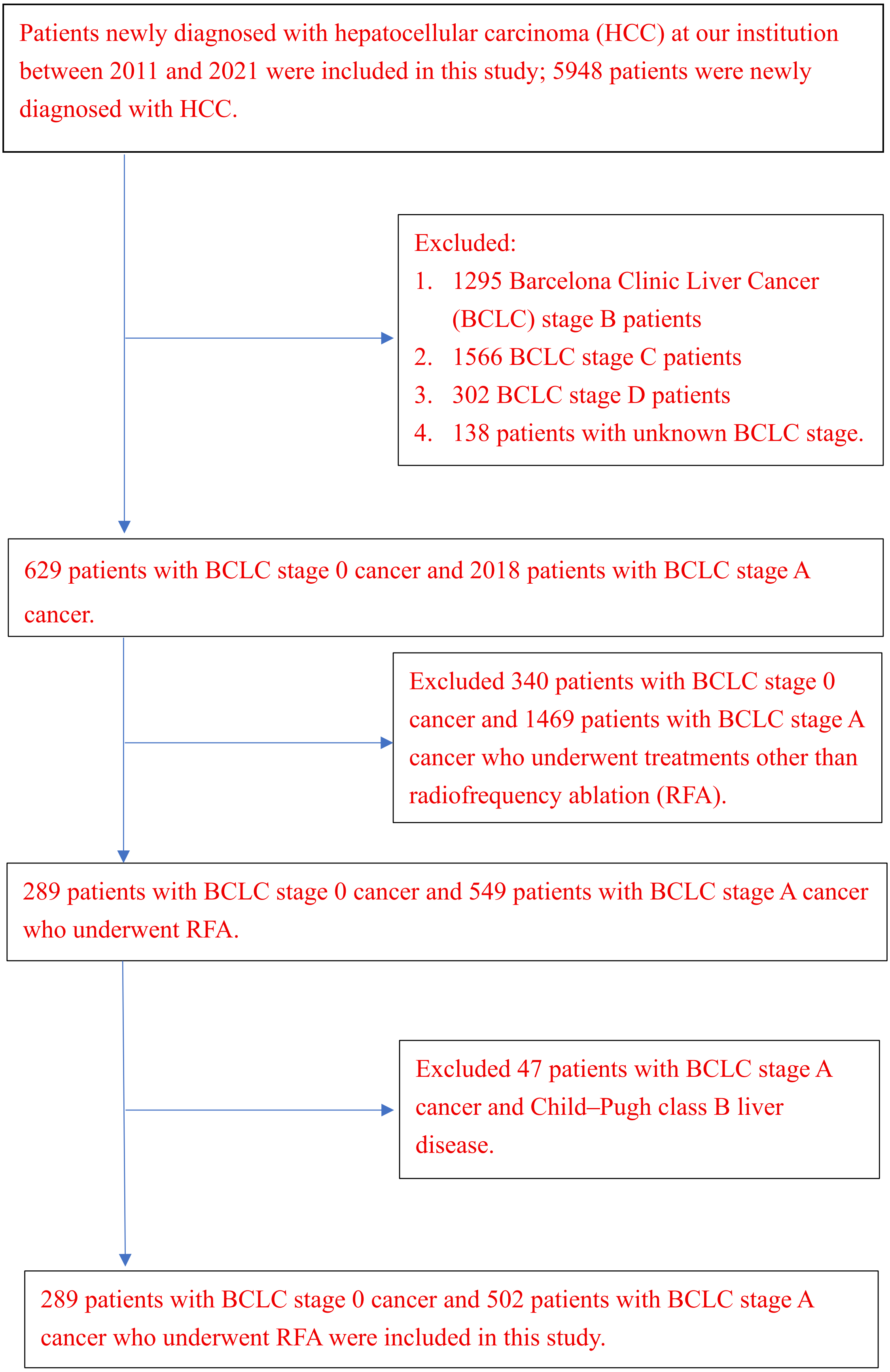
Patients newly diagnosed with hepatocellular carcinoma (HCC) at our institution between 2011 and 2021 were included in this study; 5948 patients were newly diagnosed with HCC.
Excluded:
1. 1295 Barcelona Clinic Liver Cancer (BCLC) stage B patients
2. 1566 BCLC stage C patients
3. 302 BCLC stage D patients
4. 138 patients with unknown BCLC stage.
629 patients with BCLC stage 0 cancer and 2018 patients with BCLC stage A cancer.
Excluded 340 patients with BCLC stage 0 cancer and 1469 patients with BCLC stage A cancer who underwent treatments other than radiofrequency ablation (RFA).
289 patients with BCLC stage 0 cancer and 549 patients with BCLC stage A cancer who underwent RFA.
Excluded 47 patients with BCLC stage A cancer and Child–Pugh class B liver disease.
289 patients with BCLC stage 0 cancer and 502 patients with BCLC stage A cancer who underwent RFA were included in this study.
