# Supplementary figures and images for: Predictors of True Early Recurrence in Patients Undergoing Radiofrequency Ablation for Hepatocellular Carcinoma
Source: JGH Open. 2026 Apr 3;10(4):e70400. doi: 10.1002/jgh3.70400 (PMC13051943; doi:10.1002/jgh3.70400)

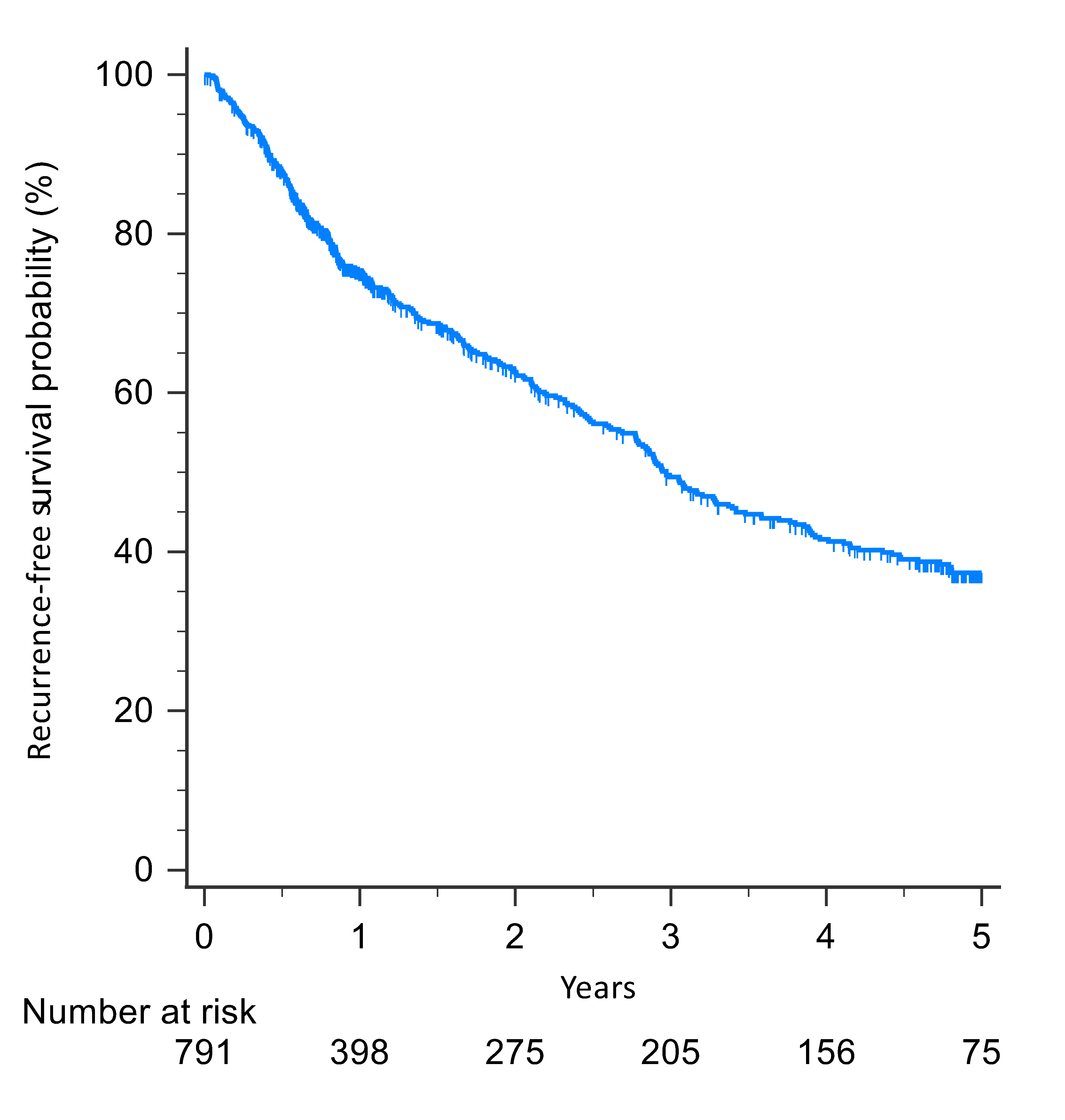

Supplement: Supplementary file 2 — Figure S2: Kaplan–Meier curves for recurrence‐free survival after excluding 33 patients who showed local tumor progression within 2 years of initial radiofrequency ablation. [file JGH3-10-e70400-s001.tif]

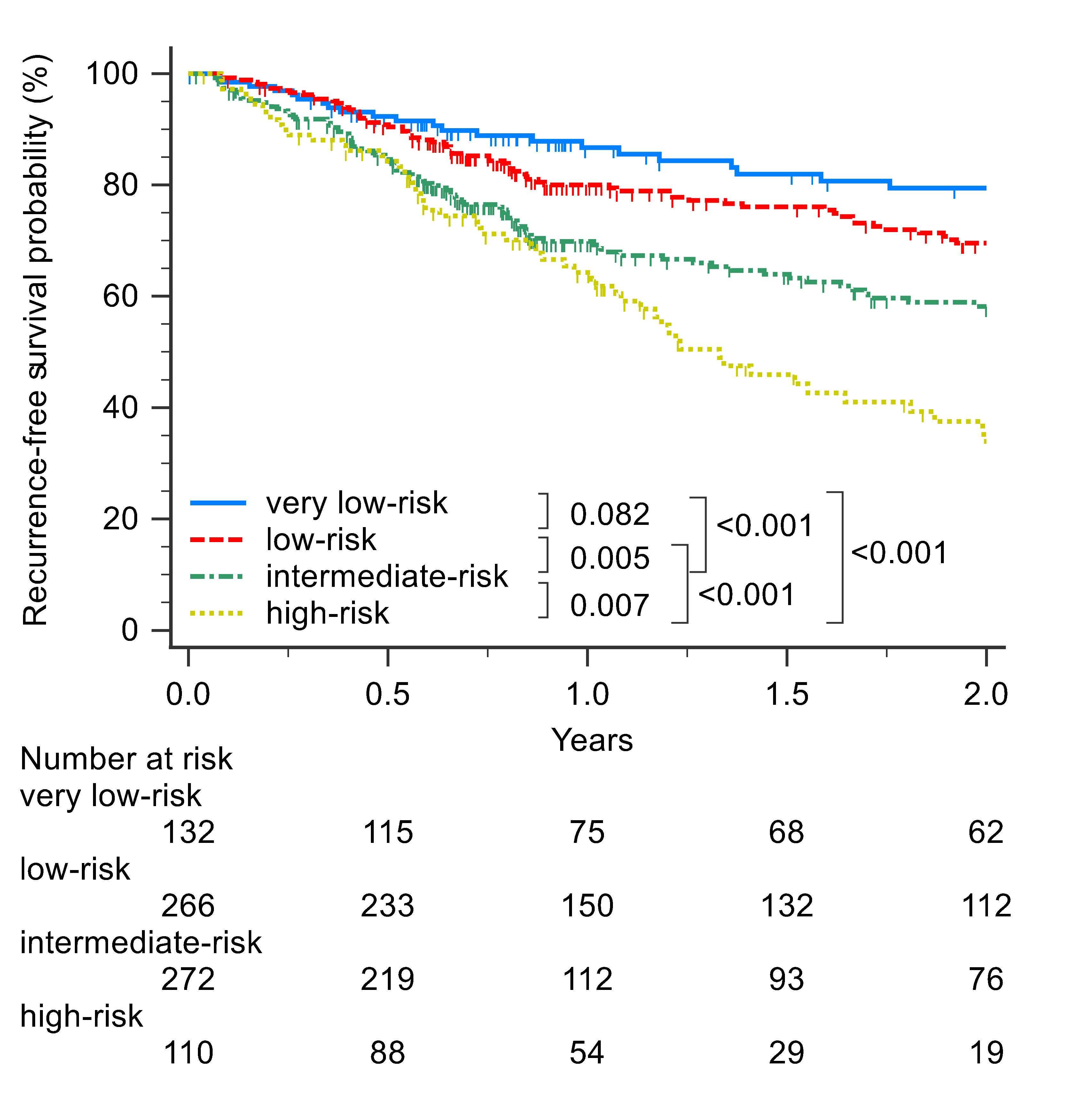

Supplement: Supplementary file 3 — Figure S3: Kaplan–Meier curves for 2‐year recurrence‐free survival stratified by four risk groups. [file JGH3-10-e70400-s003.tif]

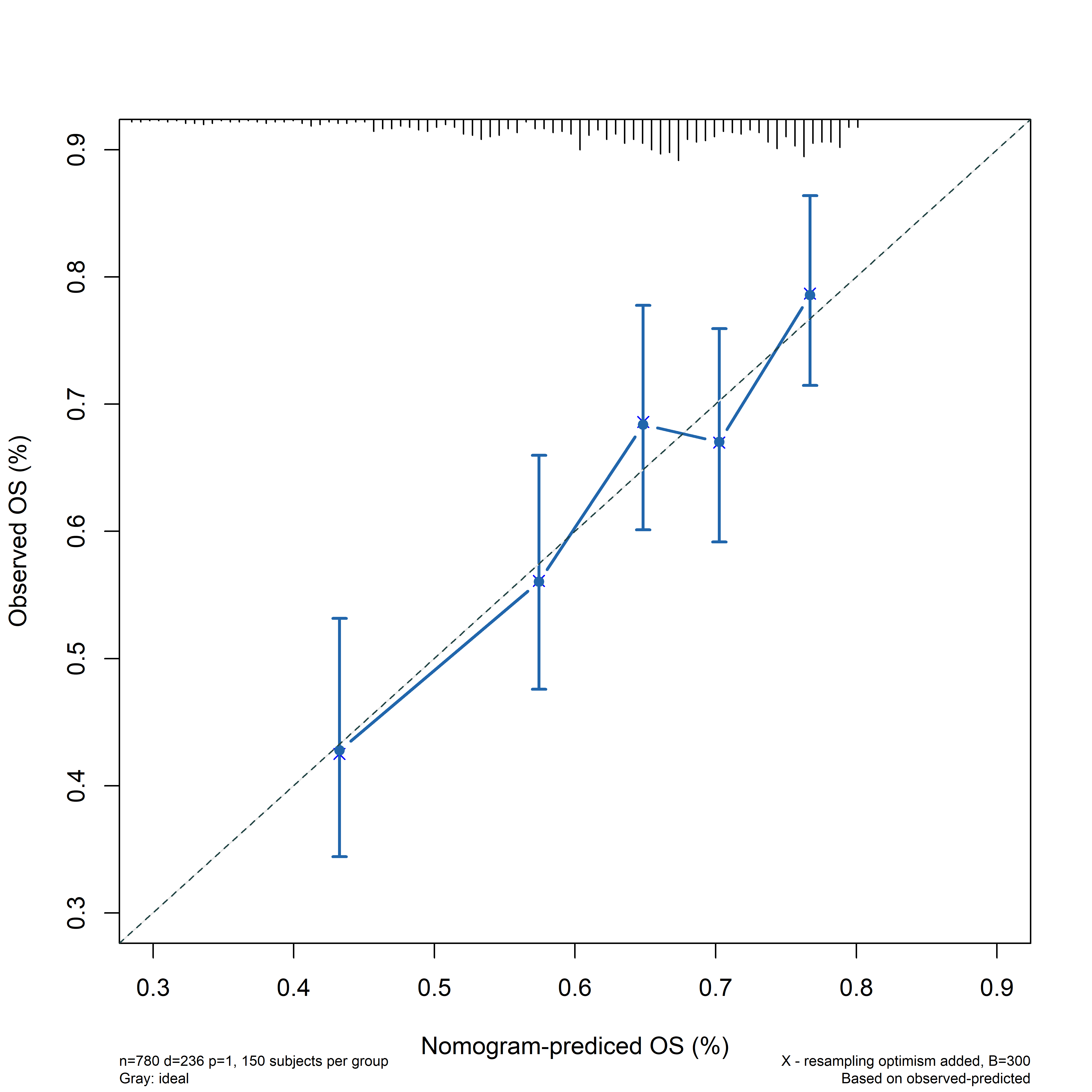

Supplement: Supplementary file 4 — Figure S4: Calibration curves of the nomogram for predicting early recurrence. [file JGH3-10-e70400-s002.tif]
